# Effects of Low-Level Autonomic Stimulation on Prevention of Atrial Fibrillation Induced by Acute Electrical Remodeling

**DOI:** 10.1155/2013/781084

**Published:** 2013-06-20

**Authors:** Yubi Lin, Ning Bian, Hairui Li, Jia Chen, Huijie Xing, Hong Li, Dandan Huang, Xianwu Lan, Bojun Gong, Li Zhou, Ruijie Liu, Min Guan, Dongdong Zhang, Gang Du, Zhengyi Huang, Xiaoming Chen, Tao Zhang, Jianyi Feng, Shaorong Wu, Liwei Wang, Aidong Zhang, Zicheng Li

**Affiliations:** ^1^Department of Cardiology, The First Affiliated Hospital of Jinan University, Guangzhou 510630, China; ^2^The Second Department of Cardiology, The Guangdong No. 2 Provincial People's Hospital, Guangzhou 510317, China; ^3^Laboratory Animal Center of Jinan University, Guangzhou 510632, China; ^4^Department of Interventional Radiology and Vascular Surgery, The First Affiliated Hospital of Jinan University, Guangzhou 510630, China; ^5^Department of Cardiothoracic Surgery, The First Affiliated Hospital of Jinan University, Guangzhou 510630, China; ^6^Department of Physiology of Medical College of Jinan University, Guangzhou 510632, China

## Abstract

*Background*. Rapid atrial pacing (RAP) can induce electrical and autonomic remodeling and facilitate atrial fibrillation (AF). Recent reports showed that low-level vagosympathetic nerve stimulation (LLVNS) can suppress AF, as an antiarrhythmic effect. We hypothesized that LLVNS can reverse substrate heterogeneity induced by RAP. *Methods and Results*. Mongrel dogs were divided into (LLVNS+RAP) and RAP groups. Electrode catheters were sutured to multiple atrial sites, and LLVNS was applied to cervical vagosympathetic trunks with voltage 50% below the threshold slowing sinus rate by **⩽**30 msec. RAP induced a significant decrease in effective refractory period (ERP) and increase in the window of vulnerability at all sites, characterized by descending and elevated gradient differences towards the ganglionic plexi (GP) sites, respectively. The ERP dispersion was obviously enlarged by RAP and more significant when the ERP of GP-related sites was considered. Recovery time from AF was also prolonged significantly as a result of RAP. LLVNS could reverse all these changes induced by RAP and recover the heterogeneous substrate to baseline. *Conclusions*. LLVNS can reverse the electrical and autonomic remodeling and abolish the GP-central gradient differences induced by RAP, and thus it can recover the homogeneous substrate, which may be the underlying mechanism of its antiarrhythmic effect.

## 1. Introduction

Atrial fibrillation (AF) is the most common cardiac rhythm disorder and contributes to thromboembolism. The presence of AF is an independent risk factor for thromboembolism; especially stroke in association with AF increases mortality and morbidity, leading to greater disability, longer hospital stays, and worse quality of life [1, 2]. AF affects approximately 10 million people in China alone [3]. AF is a complex disease that is initiated by a specific trigger and maintained in the presence of a vulnerable substrate. The autonomic nervous system (ANS), especially the vagosympathetic/vagus nerve, is an important participant in the mechanism of AF initiation.

The cardiac ANS consists of extrinsic and intrinsic components [4, 5]. The extrinsic component comprises cervical and thoracic stellate ganglia, which contribute to complex sympathetic innervation of the heart. Most of the vagosympathetic trunks of the extrinsic system contain vagal nerve fibers; they serve as parasympathetic innervation and converge at the epicardial fat pads around the superior vena cava, pulmonary veins (PVs), and the aorta, postulated to be “integration centers.” The extrinsic component modulates cardiac electrophysiology and the inducibility of AF [6, 7]. The intrinsic cardiac ANS is a complex neural network composed of ganglionated plexi (GP) concentrated within epicardial fat pads, and interconnecting ganglia and axons [8–10]. Stimulation of GP can convert the firing from PVs into AF in dogs and in humans [11–13], then promote maintenance of AF due to markedly reduced atrial effective refractory period (ERP), widen the window of vulnerability (WOV), and increase gradient differences in ERP and WOV towards GP, which serve as heterogeneous substrate caused by GP hyperactivity [14]. This mechanism is largely based on the observation that vagosympathetic nerve stimulation (VNS) can greatly facilitate initiation and maintenance of AF by activating the “integration centers of GP” [7, 15]. Once initiated, AF leads to atrial remodeling, which involves electrical and structural remodeling and even promotes autonomic remodeling; this facilitates maintenance and recurrence of AF.

Ablation of the autonomic substrate suppresses or eliminates focal AF originating from PVs [11]. In particular, GP ablation can result in successful autonomic denervation, thus prevent the induction of AF, attenuate complex fractionated atrial potentials, and suppress vagally induced AF [16–18]. Until recently, the success rate of a single or multiple circumferential PV ablations for paroxysmal AF was 70–90% (followup for about 1 year) [19], and refractory AF was reported in some of the patients. Moreover, although GP ablation significantly reduced the vagal stimulation effect, AF inducibility was strongly augmented by vagal stimulation 4 weeks after GP ablation [20]. It was also reported that, 8 weeks after ARGP and IRGP ablation, AF was induced easily by atrial RAP [21]. Therefore, the long-term effects of GP ablation are debatable. Although the autonomic effect is eliminated after GP ablation, atrial autonomic intervention remodeling and neurohormonal disturbances in the atrium may also be responsible for AF vulnerability: for example, atrial natriuretic peptide, a substrate for arrhythmia, is associated with shortened atrial conduction time and effective refractory period (ERP) [21]. Therefore, there is a need for new therapeutic strategies.

Low-level VNS (LLVNS) is being explored as another strategy. Strong-level VNS that produces >60% prolongation of the sinus cycle length facilitates AF; it produces long pauses and even sinus arrest, which are generally required to induce and maintain AF [16, 22–24]. However, moderate-level VNS (MLVNS) that produces <40% prolongation of the sinus cycle length appears not to induce AF. It has been reported that MLVNS that slowed the sinus rate by 10% for 4 weeks did not induce AF, and was not associated with arrhythmogenic risk [23]. Some recent evidence suggests that LLVNS, with voltage levels 10–50% below threshold, which does not slow the sinus rate, has an antiarrhythmic effect [25–28].

Based on all these reported results, we hypothesized that the antiarrhythmic effect of LLVNS is brought about by elimination of the heterogeneous substrate surrounding the GP areas, which is induced by rapid atrial pacing (RAP). This study was specifically designed to test the previous hypothesis in dogs with acute electrical remodeling.

## 2. Methods

### 2.1. Animal Preparation

**Figure 1:**
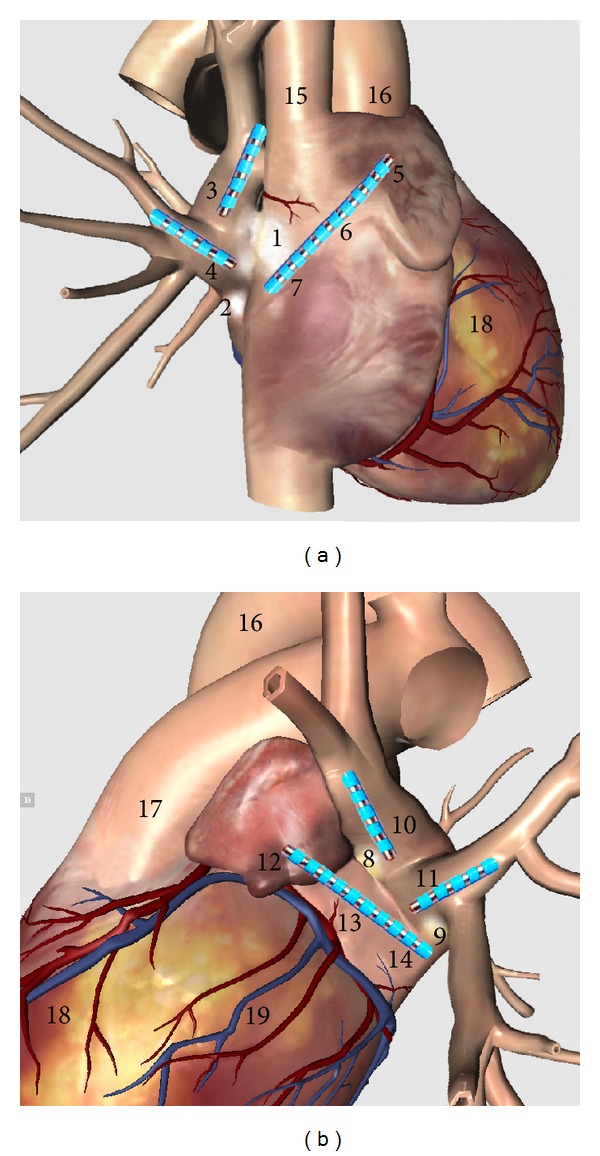
Position of catheters in the atrium. 1, anterior right ganglionated plexi (ARGP); 2, inferior right ganglionated plexi (IRGP); 3, right superior pulmonary vein (RSPV); 4, right inferior pulmonary vein (RIPV); 5, right appendages (RAA); 6, right atrium (RA); 7, margin of anterior right ganglionated plexi (ARGPM); 8, superior left ganglionated plexi (SLGP); 9, inferior left ganglionated plexi (ILGP); 10, left superior pulmonary vein (LSPV); 11, left inferior pulmonary vein (LIPV); 12, left appendages (LAA); 13, left atrium (LA); 14, Marshall ligament area (MSL); 15, superior vena cava (SVC); 16, aorta; 17, pulmonary artery (PA); 18, right ventricle (RV); and 19, left ventricle (LV).

The Institutional Animal Care and Use Committee of JINAN University of Experimental Animal Management Centre reviewed and approved the design of all animal experiments. All animal studies were reviewed and approved by the animal experimental administration of JINAN University of China. A total of 20 adult mongrel dogs weighing 13–17 kg were anesthetized with sodium pentobarbital (initial bolus, 30 mg/kg body weight, i.v.), with an additional dose of 2 mg/kg given at the end of each hour. All dogs were ventilated with room air using a positive pressure respirator, and oxygen saturation was maintained at 95–100%. The animals were fixed on an operating table, the temperature of which thermostatically controlled at 37°C. The chest was entered via bilateral thoracotomy at the bilateral fourth intercostal space. Several 10-bipolar electrodes were sutured using a noninjurious method to allow recording and stimulation at the left superior pulmonary vein (LSPV), left inferior pulmonary vein (LIPV), left appendage (LAA, catheter: LA1,2), left atrium (LA, catheter: LA5,6), area around the Marshall ligament (MSL, catheters: LA9,10) [15, 29], right superior pulmonary vein (RSPV), right inferior pulmonary vein (RIPV), right appendage (RAA, catheter: RA1,2), right atrium (RA, catheter: RA5,6), and margin of the anterior right ganglionated plexi (ARGPM, catheter: RA9,10) (Figure 1). The capture threshold of the electrodes was tested and stabilized at 1-2 V. The incisions were sutured, and then the thoracic cavity was pumped till negative pressure was achieved and closed completely.

Standard ECG and electrophysiological channels (IECG) were continuously recorded and filtered at 0.05–100 Hz and 200–1200 Hz, respectively. All tracings from the electrode catheters were amplified and digitally recorded using a computer-based electrophysiological system (Lead2000B; Jingjiang Inc., China).

### 2.2. Programmed Stimulation

In both groups, RAP was delivered (1200 bpm, 4 V, 1 msec in duration) at the LA. After each pacing hour, RAP was temporarily stopped to measure the electrophysiological data. 

Programmed stimulation was performed with Lead2000B stimulator. Regular atrial pacing as S_1_-S_1_ interval was set at 330 msec, ERP was determined using S_1_-S_2_ programmed stimulation (S_1_-S_1_ : S_1_-S_2_ = 8 : 1), which decreased from 150 msec by 10 msec decrements; as the S_1_-S_2_ intervals approached the ERP, the last S_1_-S_2_ interval increased by 8 msec, and the decrement was reduced by 1 msec again, until precise ERP was reached. ERP dispersion (ERPD) was defined as the coefficient of variation (standard deviation/mean) of the ERP at all 8 or 10 sites [30], and was calculated using MATLAB-R2008. ERPD1 was calculated for RSPV, RIPV, RA, RAA, LSPV, LIPV, LA, and LAA. When adding the ERP of ARGPM and MSL into calculation together with the previous 8 sites, we would get the values of ERPD2. 

The WOV was used as a quantitative measure of AF inducibility. During ERP measurements, if AF or >2 echo was induced by decremental S_1_-S_2_ stimulation, the difference between the longest and the shortest S_1_-S_2_ interval was designated as the WOV. The *∑*WOV was counted as the sum of WOV at each site [14].

The stimulation of the atrium was delivered at the LA site with S_1_-S_1_ intervals set at 100 msec and sustained for 10 seconds, which induced atria tachycardia or AF easily when it was continued or stopped. The recovery time (RT) was represented as the duration from AF triggered by persistent S_1_-S_1_ stimulation to sinus rhythm.

### 2.3. Low-Level Vagosympathetic Nerve Stimulation

**Figure 2:**
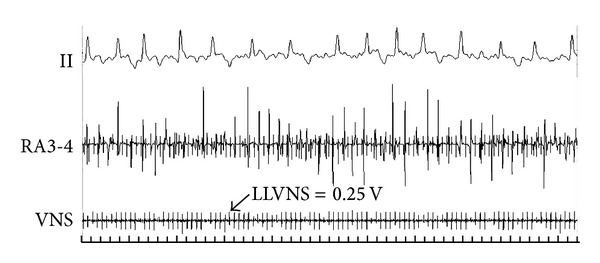
Low-level vagosympathetic nerve stimulation (LLVNS) during rapid atrial pacing (RAP). The voltage of LLVNS was 0.25 V (VNS channel). RAP was delivered at the LA site. RA3,4 channel recorded the signal of RAP and LLVNS.

Bilateral cervical vagosympathetic trunks were decentralized by surgical procedures. Two pairs of electrodes insulated with surrounding tissue were embedded in the vagosympathetic trunks located adjacent to the cervical artery for LLVNS [31]. LLVNS was induced by applying high-frequency electrical stimulation (HFS, 20 Hz, 0.1 msec in duration, square waves, voltage 0.1–0.5 V) to vagosympathetic nerves via an electronic stimulator (BL-420E Experimental System; Tai Meng, Chengdu, China) (Figure 2). The lowest voltage of VNS that prolonged the cycle length of sinus rhythm (A-A intervals) by no more than 30 msec was considered the threshold. The LLVNS voltage was approximately 50% below the threshold. Prior to each hour of LLVNS, the threshold of VNS was determined again to adjust the LLVNS voltages for the next hour [25, 26, 28].

### 2.4. Experimental Protocol

Twenty dogs were randomly divided into the experimental group (*n* = 10), which underwent RAP remodeling concomitant with LLVNS for 3 hours (Figure 2), and the control group (*n* = 10), which only underwent RAP remodeling for 3 hours. In the experimental group, LLVNS was delivered to bilateral cervical vagosympathetic nerves by applying HFS as described previously. The ERP and WOV were measured at LSPV, LIPV, LA, LAA, MSL, RSPV, RIPV, ARGPM, RA, and RAA at baseline levels and the end of every hour during the 3 hours pacing period.

### 2.5. Statistical Analysis

All values were expressed as the mean ± standard deviation of the mean. Paired *t*-test was used for comparisons of ERP and WOV of baseline levels and each pacing period. Analysis of variance (ANOVA) by SPSS20 was used to compare ERP, WOV, ERP dispersion, and RT in both groups, and graphs were drawn with Microsoft Excel2010. *P* values ≤0.05 were considered to indicate statistical significance.

## 3. Results

The systolic and diastolic blood pressures were stable during the entire experimental period, with no sign of heart failure throughout. Oxygen saturation was maintained at 95–100% during the experiments.

### 3.1. Effective Refractory Period

**Figure 3:**
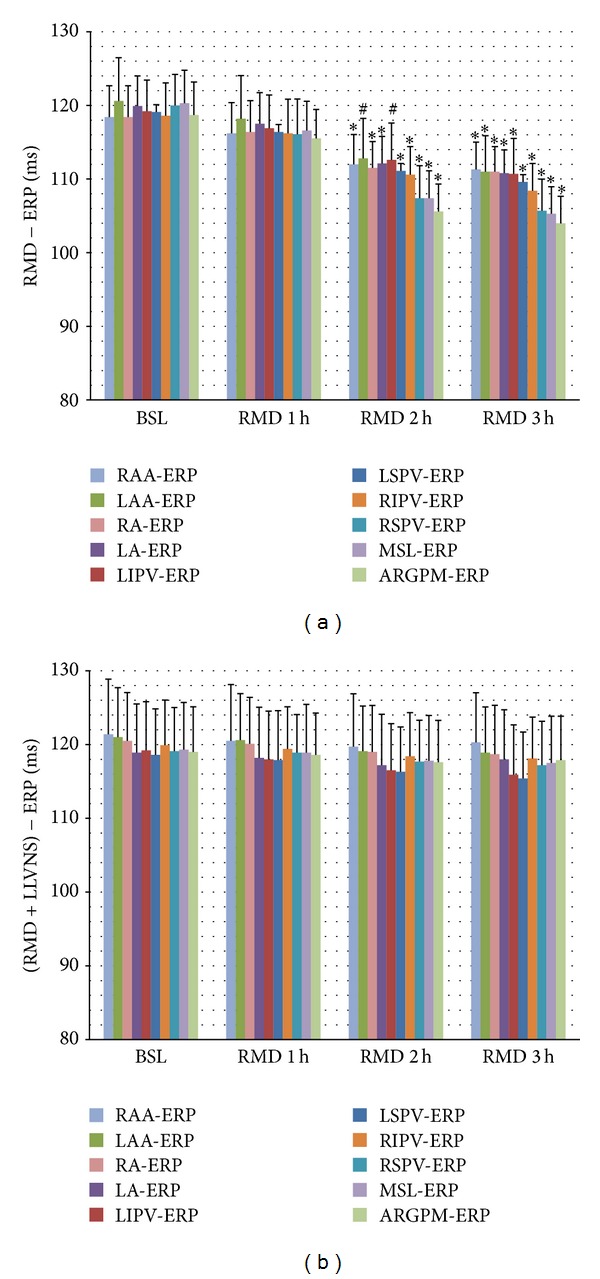
Effects of LLVNS on ERP and its gradient. LLVNS reversed the decrease in ERP and increase in ERP gradient that was induced by RAP. ^#^ *P* < 0.05 and *P < 0.01 compared with baseline levels using the paired *t*-test. ERP: effective refractory period; BSL: baseline level; RMD: remodeling; LLVNS: Low-level vagosympathetic nerve stimulation.

In the control group, the ERP at RSPV, RIPV, RA, RAA, ARGPM, LSPV, LIPV, LA, LAA, and MSL was markedly shortened in the second and third hour (Figure 3). The decrease in ERP at ARGPM, MSL, RSPV, RIPV, and LSPV was more significant compared to other sites of the atrium. Moreover, there were apparent ERP gradients in the atrium at the second and third hour. 

In contrast, in the experimental group, the ERP of each site of the atrium showed no significant variance at the end of each hour, compared to baseline levels. Moreover, ERP gradients were not observed at the end of each hour, as observed at the baseline.

### 3.2. Window of Vulnerability

**Figure 4:**
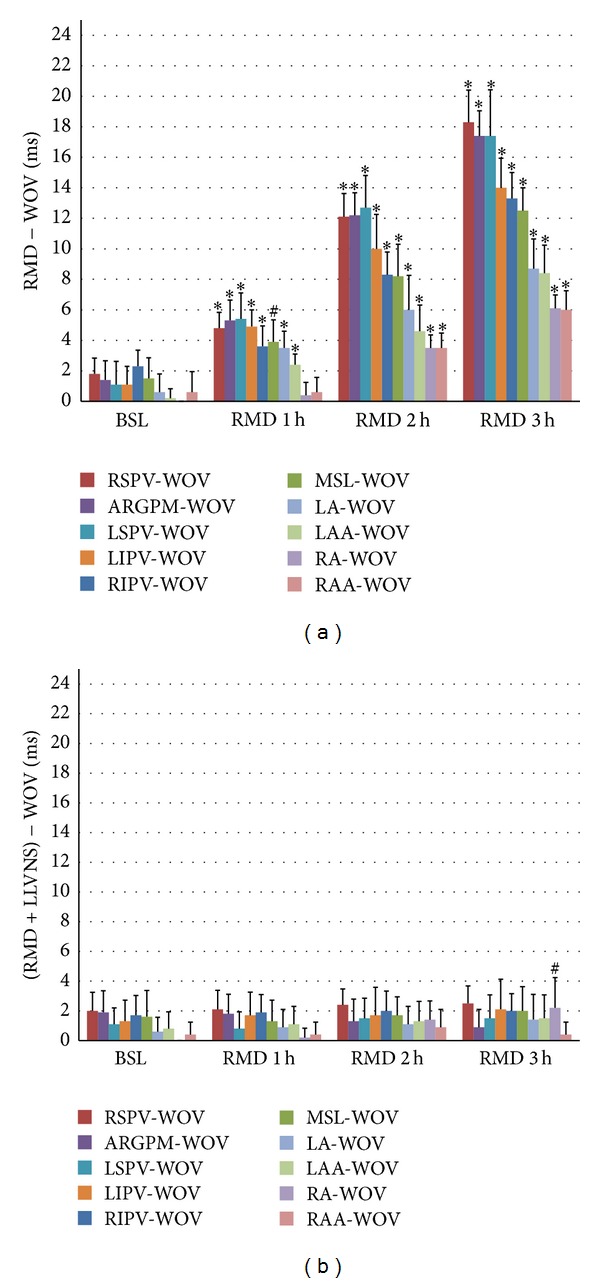
Effects of LLVNS on WOV and its gradient. LLVNS reversed the increase in WOV and its gradient caused by RAP. WOV: window of vulnerability; the remaining symbols and abbreviations are the same as those used in Figure 3.

In the control group, the WOV at each site of the atrium showed a significantly progressive increase during each hour of RAP (Figure 4). Moreover, a significant WOV gradient was observed in the atrium, and the WOV progressively increased across sites in the given order: RAA and RA, LAA, LA, MSL, RIPV, LIPV, LSPV, ARGPM, and RSPV.

In the experimental group, the WOV at each site of the atrium was not different between each period of RAP, except for RA at the third period (*P* < 0.05), compared to the baseline value; also, there was no significant WOV gradient at each site of the atrium.

### 3.3. Dispersion of the Effective Refractory Period

**Figure 5:**
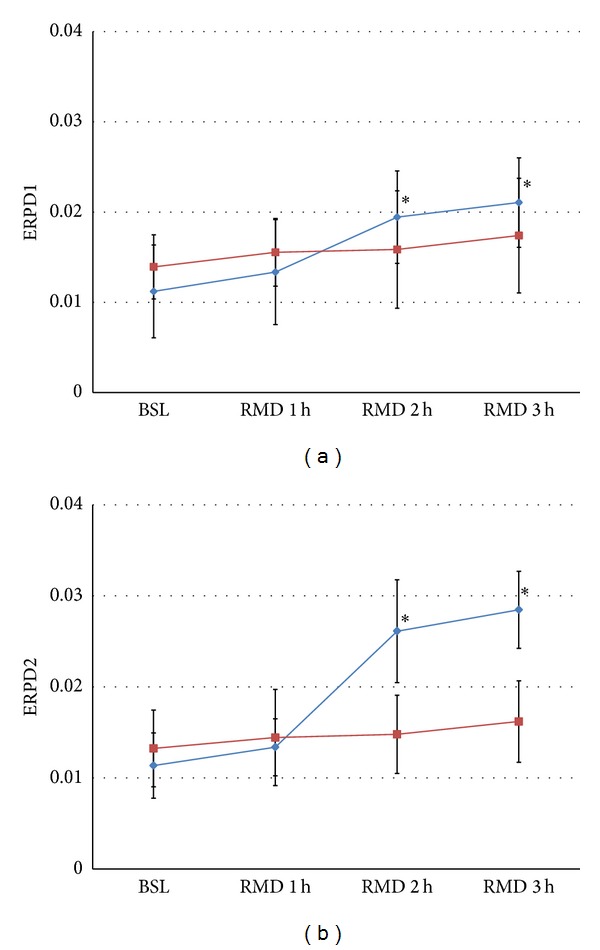
Effects of LLVNS on ERP dispersion. LLVNS reversed the effects of RAP on ERP dispersion. The blue (⋄) and red (□) curves represent the control group and experimental group, respectively. The symbols and abbreviations are the same as those in Figure 3.

In the control group, ERPD1 and ERPD2 showed progressive increase and reached statistical significance in the second and third hour of RAP (Figure 5). In contrast, the experimental group showed no significant difference in ERPD1 and ERPD2 for each RAP period. Moreover, ERPD2 was more obviously enlarged than ERPD1 in the control group, but there was no significant difference between ERPD1 and ERPD2 in the experimental group during each period. 

### 3.4. Recovery Time and Low-Level Voltage

**Figure 6:**
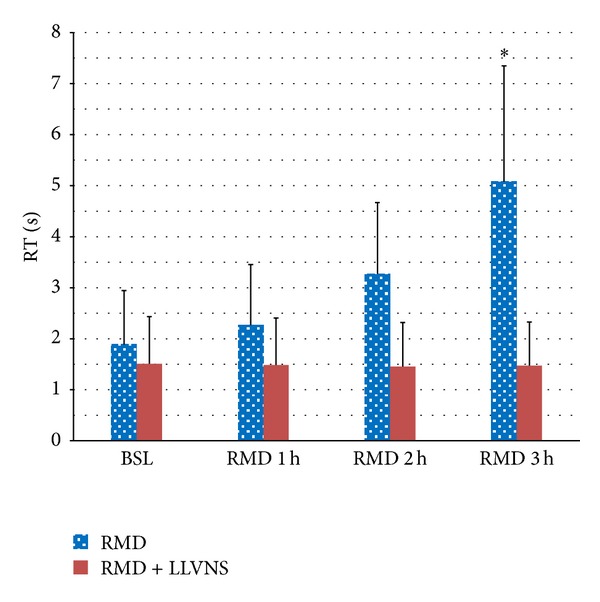
The effect of LLVNS on recovery time. The recovery time (RT) was represented as the duration from AF triggered by persistent S_1_-S_1_ stimulation to sinus rhythm. Abbreviations are the same as those in Figure 3.

**Figure 7:**
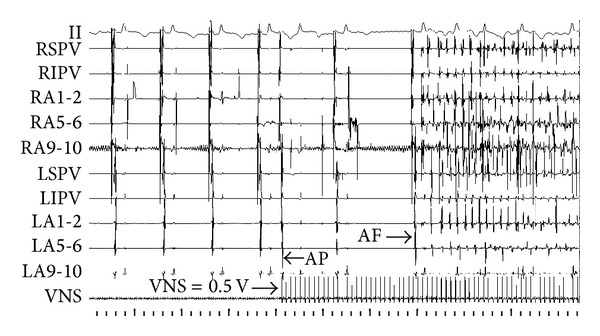
Vagosympathetic nerve stimulation (VNS) promoted atrial arrhythmia. VNS with the voltage at 0.5 V triggered atrial premature and then facilitated AF. AP: atrial premature. AF: atrial fibrillation.

**Figure 8:**
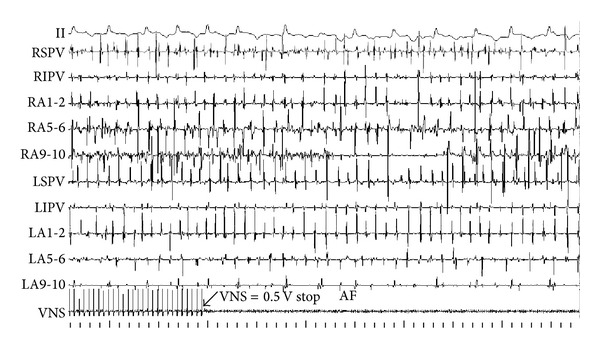
VNS maintained AF. The AF was persistent during VNS with the voltage at 0.5 V. While the VNS was removed, AF still maintained. AF: atrial fibrillation.

**Figure 9:**
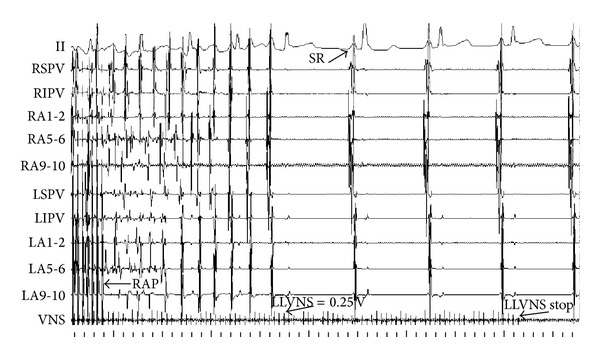
LLVNS cannot maintain the AF. When RAP that is persistent for 3 hours concomitant with LLVNS (0.25 V), was stopped, the AF progressively terminated even though the LLVNS still continued. RAP: rapid atrial pacing. SR: sinus rhythm.

The recovery time (RT) was represented as the duration from atrial arrhythmia that is facilitated by fast pacing triggers in atrium to sinus rhythm. In control group, RAP remodeling progressively increased RT (Figure 6). While RAP was persistent for 3 hours, the RT was obviously longer than that of baseline (5.078 ± 2.266 seconds versus 1.886 ± 1.059 seconds, *P* < 0.01). In experimental group, combining RAP with LLVNS for 3 hours, the RT at each period has no difference from that of baseline levels. During the judgments of threshold, the voltage at 0.5 V that slowed the sinus rate apparently can lead to atrial premature and then facilitate AF at baseline (Figure 7). The AF was persistent during the VNS. While the VNS was removed, AF still maintained (Figure 8). When RAP that is persistent for 3 hours concomitant with LLVNS (0.25 V) was stopped, the AF progressively terminated even though the LLVNS still continued (Figure 9). The low-level voltages for LLVNS that is 50% below the threshold that prolonged the A-A intervals of sinus rhythm by no more than 30 msec had not changed, prior to the first, second, and third remodeling (Pre-RMD 1 h, 0.154 ± 0.058 V; Pre-RMD 2 h, 0.22 ± 0.071 V; Pre-RMD 3 h, 0.225 ± 0.115 V, *P* > 0.05).

## 4. Discussion

We have successfully proved our hypothesis about the benefits of LLVNS for treating AF. This is clear from the results, which show that application of LLVNS to canine models of RAP remodeling markedly reversed the decrease in ERP and increase in WOV at each site. Moreover, it abolished gradient differences in the electrophysiological substrate surrounding GP and thus maintained ERP dispersion at baseline levels. It also reduced the RT from AF. 

In our research, as we measured the threshold, the VNS with the voltage at 0.5 V triggered atrial premature and then facilitated AF at baseline. The AF was persistent during the VNS. While the VNS was removed, AF still maintained. These results suggest that vagosympathetic trunks as extrinsic components play an important role in the initiation and maintenance of AF. The arrhythmic effect of VNS may be mediated by activating the “integration centers of GP” [7, 15]. 

 The hyperactivity of the autonomic element in MSL may contribute to the initiation of AF and even ventricular tachyarrhythmia [32]. It is reported that MSL participates in the interaction between integration centers and GP [15]. High-level stimulation of GP can progressively reduce atrial ERP, widen the WOV, and lead to gradient differences in ERP and WOV towards GP [14], and it is even associated with complex fractionated atrial potentials [33]. Similar to these reports, in our study too, RAP remodeling for 2-3 hours led to a significant decrease in ERP and caused an obvious decrease in the ERP gradient from the appendages towards PV, MSL, and ARGP, which serve as GP areas. Moreover, ERP dispersion progressively increased and reached a significant difference at the end of RAP, and when the ERP dispersion values for ARGP and MSL were added, ERP dispersion was further augmented. The WOV progressively increased during RAP, and it was noted that the increase in WOV at PV, MSL, and ARGP was more apparent, which caused an increase in the gradient difference from the non-GP area towards the GP sites. Based on all these results, we concluded that the ERP and WOV gradients were reflective of the electrophysiological substrate surrounding GP, since autonomic remodeling is characterized by hyperactive GP caused by RAP.

In the RAP remodeling, when electrical remodeling occurs concomitantly with autonomic remodeling, it is believed to indicate progressive enhancement of neural activity [34]; in this study, heterogeneous differences in both ERP and WOV were observed at atrial sites, especially around the GP, so it is possible that RAP resulted in obvious hyperactivity of GP and thus increased vulnerability to AF because of the increased heterogeneity of the substrate. 

It has been reported that LLVNS that is 10–50% below the threshold voltage, required to slow the sinus rate or atrioventricular conduction, may prevent episodic AF caused by rapid PV and non-PV firing, due to a progressive increase in AF threshold at all PVs and atrial appendages sites, particularly RSPV, RIPV, LSPV, and RAA. Moreover, this type of antiarrhythmic effect is not dependent on the activation of the afferent vagal nerve fibers that project to the brain [28]. The activation of neural elements within the ARGP, SLGP, and stellate ganglion was attenuated by LLVNS application to both [28, 35] or one vagosympathetic trunk [26, 27], auricular branch of the vagus nerve of the right ear (low-level tragus stimulation) [36], or superior vena cava [34]. Therefore, the antiarrhythmic effect of LLVNS may be brought about via its action on GP sites. When the voltage of LLVNS was further reduced to 80% below the threshold, it still induced a similarly antiarrhythmic effect [36]. LLVNS at these values eliminated the spatial gradient of ERP and WOV induced by ARGP stimulation from the PV-atrial junction toward the atrial appendage [25]. Moreover, it could prevent and reverse atrial remodeling induced by RAP as well as suppress AF induced by strong cholinergic stimulation, for example, by injection of acetylcholine (10 mM) into the ARGP or RAA. The duration and cycle length of AF decreased obviously during LLVNS [35]. In contrast, stimulation of ARGP and SLGP for 6 hours with a voltage that causes a 10% decrease in sinus rate does not appear to have an antiarrhythmic effect [37]. In our study, we used LLVNS values at 50% below the threshold (which slowed the sinus rate by no more than 30 msec). We found that this reversed the electrical and autonomic remodeling, restoring ERP and WOV to baseline levels. LLVNS maintained the two kinds of ERP dispersion at baseline levels. Also, the ERP and WOV around ARGP, PV, and MSL were not significantly different from those in other areas. Thus, LLVNS resulted in substrate homogeneity. Thus, they are in agreement with previously published results. Based on our current study and previous studies, we think that hyperactivation of intrinsic nerves or “integration centers” was suppressed by LLVNS. It brought about this effect by abolishing gradient differences in the substrate surrounding GP, which contributed to the homogeneity of ERP and WOV in the atrium. More importantly, the duration of AF promoted by fast-pacing triggers at the end of each period was significantly decreased. During the process of RAP and LLVNS at the third period, when the RAP was removed, the AF induced by RAP recovered to sinus rhythm immediately, even though LLVNS was still continued.

Based on our results, the mechanism of action of LLVNS may be explained as follows: (1) suppression of the neural activity of GP, which abolishes gradient differences in the substrate surrounding GP and (2) enhanced recovery of the homogeneous substrate with baseline susceptibility to AF. In addition, there are other mechanisms that have been proposed by researchers: (3) decrease in transient intracellular Ca^2+^ levels, owing to reduced release of sympathetic neurotransmitters via inhibition of activity of the stellate ganglion [27, 38], (4) desensitization of autonomic receptors, such as *β*-adrenergic receptors [25, 39, 40], (5) increase in nitric oxide availability mediated by upregulation of the PI3K/NO signaling pathway [41], which subsequently leads to inhibition of GP function, (6) increase in the levels of neuropeptides or neurotransmitters inhibiting GP function, for instance the neuropeptide Y2 and vasostatin-1, which exert strong antiadrenergic and anticholinergic effects [41–43]. Future studies should be conducted to integrate and explain these mechanisms in relation to one another.

A limitation of this study is that we have no direct evidence indicating inhibition of neuronal firing within GP and MSL, and the results are therefore limited to short-term RAP and LLVNS. This hypothesis will have to be verified in the future by using RAP and LLVNS of longer duration. This will help evaluate the antiarrhythmic effects of LLVNS in chronic AF models. Moreover, research will be required to determine the optimal parameters and sites for LLVNS that will have the greatest degree of AF inhibition with minimal side effects. We hope to devise future treatment methods to treat chronic AF, in which autonomic nerve stimulators can be inserted using noninvasive methods.

## 5. Conclusions

LLVNS can reverse the electrical and autonomic remodeling induced by RAP. The mechanism involves abolishing gradient differences in the substrate surrounding GP and recovery of the homogeneous substrate.
